# Supplementary material for: Radiation therapists’ perceptions of thermoplastic mask use for head and neck cancer patients undergoing radiotherapy at Ocean Road Cancer Institute in Tanzania: A qualitative study
Source: PLoS One. 2023 Feb 23;18(2):e0282160. doi: 10.1371/journal.pone.0282160 (PMC9949626; doi:10.1371/journal.pone.0282160)
Supplement: S1 File — (ZIP) [file pone.0282160.s001.zip › Supporting information/INTERVIEW GUIDE.docx]

**INTERVIEW GUIDE**

**Objective:** To explore Radiation Therapists’ perceptions of thermoplastic mask use for head and neck cancer radiotherapy

**Introduction**

Thank you very much for agreeing to participate in this interview. The purpose of this study is to explore Radiation therapists’ perceptions about thermoplastic mask use as an immobilization device for head and neck cancer patients at Ocean Road Cancer Institute. You have been requested because you working here and have vast experience in thermoplastic mask use for head and neck cancer radiotherapy. During this interview there are no wrong answers, all answers are accepted so kindly feel free. I would like to remind you that, this interview will take not more than 30 minutes and it will be recorded to grab points that could be difficult to write down but also for easy analysis of the information later on. Your participation in this study is voluntary and you have a right to withdraw from the study at any time.

Let us begin with a few questions about you (Socio-demographics information).

| 1. Age________ 2. Gender_______ 3. Level of education____________ 4. Marital Status_________ 5. Working experience_______ |
| --- |

Let me now ask you questions regarding your perceptions on thermoplastic mask use for head and neck cancer radiotherapy.

1. What are the benefits of using a thermoplastic mask for head and neck cancer patients undergoing radiotherapy? (Probe: during patients’ setups, during pre-treatment preparation)
2. What are the limitations of using a thermoplastic mask for head and neck cancer patients undergoing radiotherapy? (Probe: Recycling of thermoplastic masks, Long waiting time between treatment preparation and delivery)
3. What are the perceived challenges that you have faced when using the thermoplastic mask as an immobilization device for HNC patients in your working place? (Probe questions: Storage, hygiene, positioning issues during patients’ setup, staff competencies on preparing thermoplastic masks, Is it a challenge to remould the used masks?)
4. What are the perceived possible solutions?
5. Is there anything else that you want to share with me about what we have just discussed?

**Thank you for your time**
