# Supplementary material for: Radiation therapists’ perceptions of thermoplastic mask use for head and neck cancer patients undergoing radiotherapy at Ocean Road Cancer Institute in Tanzania: A qualitative study
Source: PLoS One. 2023 Feb 23;18(2):e0282160. doi: 10.1371/journal.pone.0282160 (PMC9949626; doi:10.1371/journal.pone.0282160)
Supplement: S1 File — (ZIP) [file pone.0282160.s001.zip › Supporting information/Selected quotes from Transcripts.docx]

**Radiation Therapists’ perceptions of thermoplastic mask use for head and neck cancer patients undergoing radiotherapy at Ocean Road Cancer Institute in Tanzania: A qualitative study**

**Sociodemographic characteristics data**

| **Characteristic** | **Frequency** |
| --- | --- |
| Age |  |
| Between 27 and 49 | 15 |
| Level of Education |  |
| Bachelor degree | 15 |
| Marital Status |  |
| Single | 4 |
| Married | 11 |
| Professional experience |  |
| ≤5 | 9 |
| >5 | 6 |

**Excerpts of the transcripts**

**Perceived benefits and limitations**

*“That device is used to maintain a patient in a fixed position to ensure the correct delivery of radiation dose to the intended area and it is easy to construct. In our department, the patient can come today, you construct a thermoplastic mask today and start using it today during treatment”. (RTT 6)*

*“...the more you recycle (remould) thermoplastic mask, the more it loses its quality and increases the possibility of geometrical misses and become unfit to the patient”. (RTT 3)*

*“We have been getting complaints from the patients that the thermoplastic mask compresses or is unfit than it’s supposed to be”[..].“Usually more than two weeks is enough to lose its immobilization status and it starts compressing the patient or becoming unfit”. (RTT 2)*

*“Avoiding reuse of thermoplastic mask more than two times” (RTT1)*

*“A thermoplastic mask is a device used to immobilize head and neck cancer patients during treatment” (RTT 9)*

**Refresher training and supervision requirements for effective use**

*“I think what is best first is to have continuing medical education for staff and refresher training. So if there is frequent refresher training on properly using a thermoplastic mask, it will help remind the staff of its proper use”. (RTT 1)*

*“…….the big issue is lack of supervision because if it’s basic training every staff has got that. What is important is proper supervision in implementing protocol or standard operating procedures”. (RTT 12)*

**Proper storage for quality maintenance**

*“To my understanding, it is required to have sufficient storage shelving which helps to properly keep thermoplastic masks, but in our setting, we don’t have enough storage shelving. What we do is put the thermoplastic mask on top of one another. There is a possibility that the one which is beneath others gets deformed. If this happens, it becomes unfit for the planned patient”. (RTT 9)*

*“Searching for the thermoplastic mask is the main challenge, there should be a proper arrangement by having a logbook for easy identification”. (RTT 5)*

*“We put thermoplastic masks on top of the other rather than hanging as advised”.(RTT 3)*

*“Putting a thermoplastic mask on top of one another may transmit infections from one patient to another. This is the main challenge in our department because we don’t have enough storage system and room. So having an improved storage system will improve the quality of masks” (RTT 15)*

**Increased financial support and proper budgeting**

*“Increase a budget that will enable us all the time to have new thermoplastic masks instead of re-moulding used thermoplastic masks which are inefficient”. (RTT 7)*

*“The shortage of thermoplastic masks in the department is caused by a lack of funds to procure new thermoplastic masks. This results in the recycling of masks” (RTT 14)*
