# Supplementary material for: Radiation therapists’ perceptions of thermoplastic mask use for head and neck cancer patients undergoing radiotherapy at Ocean Road Cancer Institute in Tanzania: A qualitative study
Source: PLoS One. 2023 Feb 23;18(2):e0282160. doi: 10.1371/journal.pone.0282160 (PMC9949626; doi:10.1371/journal.pone.0282160)
Supplement: Attachment — Submitted filename: Response to Reviewers.docx [file pone.0282160.s002.docx]

4^th^ November 2022

Editor-in-Chief

PLOS ONE.

Dear Editor,

I would like to resubmit the enclosed manuscript ‘**Radiation therapists’ perceptions of thermoplastic mask use for head and neck cancer patients undergoing radiotherapy at Ocean Road Cancer Institute in Tanzania: A qualitative study’** for your consideration for publication in the journal of PLOS ONE after responding to each point raised by the academic editor and reviewers.

A summary of the corrections made to each point raised by the academic editor and reviewers is shown in the table below.

| **S/N** | **Comments: Academic editor** | **Authors response** |
| --- | --- | --- |
| 1. | Please ensure that your manuscript meets PLOS ONE’s style requirements, including those for file naming. The PLOS ONE style templates | Thank you. This has been corrected in a revised manuscript |
| 2. | Please provide additional details regarding participant consent. | Thank you. Additional details have been added in a revised manuscript |
| 3. | In your Data Availability statement, you have not specified where the minimal data set underlying the results described in your manuscript can be found. | Thank you. This has been specified |
| 4. | Your ethics statement should only appear in the Methods section of your manuscript. | Thank you. An ethical statement has been moved to the methods section |
|  |  |  |
|  | **Comments: Reviewer 1** |  |
|  | Congratulations with submission of the article Radiation therapists’ perceptions of thermoplastic mask use for HNC - A qualitative study. The article describes an important issue regarding the perception of radiation therapists and the use of thermoplastic masks. Some minor changes: | Thank you so much. |
| 1. | line 104: "patients" should be replaced by "RTT" or " participants" | Thank you for this observation |
| 2. | Could you suggest more practical things that could be improved e.g. giving education, 5 times a year? creating protocols? What would be the next step? | Thank you. This has been added in the conclusion section |
| 3. | For future: It would be interesting to see a follow-up of the situations, have things improved? what interventions work and what do not? | Thank you. We will make a follow-up. |
|  | Overall, an important and interesting topic. | Thank you for your positive overall impression of our manuscript |
|  |  |  |
|  | **Comments: Reviewer 2** |  |
| 1. | The objective should have been better defined. | Thank you. This has been revised |
| 2. | Academic training and the number of years of professional experience should be included. | Thank you. This has been included |
| 3. | It does not mention who carried out the interviews and content analysis. | Thank you. This has been added to the data collection and analysis section |
| 4. | The conclusions are in part aligned with the objective. | Thank you. This has been revised. |
| 5. | The study is limited to a single location, which makes this a case study, with all the limitations that this type of study entails. | Thank you for your comment. It is true this is a major limitation for the qualitative studies whose results were not meant for generalizability but rather to explore in-depth views of the study participants about the thermoplastic mask use in the study setting. However, the findings could be transferred to settings with a similar context. |
|  |  |  |
|  | **Comments: Reviewer 3** |  |
| 1. | The article „Radiation therapists’ perceptions of thermoplastic mask use for head and neck cancer patients undergoing radiotherapy at Ocean Road Cancer Institute in Tanzania: A qualitative study” is interesting especially due to its setting in a low-resource setting. However, I think the novelty of the study should be emphasized, especially since the use of thermoplastic masks in ENT cancers has become standard. Additionally, I have several questions/comments, listed below. | Thank you for your comment. We have tried to work on it |
| 2. | Who designed the questionnaire? How were the questions chosen? | The questionnaire was designed by the authors based on clinical experience with a high workload setting and limited resources. The statement has been added to the revised manuscript |
| 3. | The authors refer to RTTs as radiation specilists. Does this mean they are all physicians? Or were there also nurses and physicists involved in the study? | Thank you for this good question. RTTs are not physicians, they are technologists working in a radiotherapy department. They are neither nurses nor physicists |
| 4. | Also in the Material and Method section, I think more details should be offered. How were the data analyzed? What are the six steps used for analysis? Because to me it seems that only a summary of the interviews is provided | Thank you for this comment. This has been well explained in a revised manuscript |
| 5. | The authors refer to the concept of recycling the thermoplastic masks. I think more details should be offered on the subject, especially since this is not standard | Thank you. More details have been offered in a study setting section |
| 6. | In the Discussions section, the authors state that „This study revealed significant differences in participants’ understanding of the thermoplastic mask preparation process.” Where was this detailed in the Results section? | Thank you for pointing out this error. This sentence has been corrected. |
| 7. | Minor comments Please revise: „This is a perforated plastic sheet that is soaked in warm water which then becomes soft and flexible, moulded on the patient’s face, cools after a certain time and becomes hard and finally keeps a final shape of the patient’s anatomy (6,7)”, „During thermoplastic mask preparation, RTT explains in detail the procedure to the patient, position the patient, and perform quick and accurate construction of thermoplastic mask by adhering to the manufacturer’s guidelines while working as a team and maintaining proper documentation (9).”, „This is because of avoiding its loss, easy identification during treatment, shape maintenance and prevention of infection transmission.” | Thank you so much. We agree that these sentences are not clear. These sentences have been revised |
|  |  |  |
|  | **Comments: Reviewer 4** |  |
| 1. | Study seems to address the key routine issues and conducted well. However,I have few queries as below | Thank you |
| 2. | Experience and Knowledge level of the RTTs involved (Aged 27-49 years) is missing | Thank you. This has been added |
| 3. | internal consistency reliability test and coherence of the Thematic analysis is missing | Thank you. This has been added |

To our knowledge, we have addressed all the comments and questions raised by the reviewers. We, therefore, kindly ask you to reconsider our manuscript for publication in this very good journal.

Yours Sincerely

Furahini Yoram,

Corresponding author.

Email: [furahiniyoram@gmail.com](mailto:furahiniyoram@gmail.com)

Contact: +255756470122
