# Supplementary material for: Radiation therapists’ perceptions of thermoplastic mask use for head and neck cancer patients undergoing radiotherapy at Ocean Road Cancer Institute in Tanzania: A qualitative study
Source: PLoS One. 2023 Feb 23;18(2):e0282160. doi: 10.1371/journal.pone.0282160 (PMC9949626; doi:10.1371/journal.pone.0282160)
Supplement: Attachment — Submitted filename: Response to Reviewers - 2.docx [file pone.0282160.s003.docx]

6^th^ December 2022

Editor-in-Chief

PLOS ONE.

Dear Editor,

I would like to resubmit the enclosed manuscript ‘**Radiation therapists’ perceptions of thermoplastic mask use for head and neck cancer patients undergoing radiotherapy at Ocean Road Cancer Institute in Tanzania: A qualitative study’** for your consideration for publication in the journal of PLOS ONE after responding to each point raised by the academic editor and reviewers.

A summary of the corrections made to each point raised by the academic editor and reviewers is shown in the table below.

| **S/N** | **Journal requirements** | **Authors response** |
| --- | --- | --- |
| 1. | Please review your reference list to ensure that it is complete and correct. If you have cited papers that have been retracted, please include the rationale for doing so in the manuscript text, or remove these references and replace them with relevant current references. Any changes to the reference list should be mentioned in the rebuttal letter that accompanies your revised manuscript. If you need to cite a retracted article, indicate the article’s retracted status in the References list and also include a citation and full reference for the retraction notice. | Thank you. All the references have been reviewed |
|  |  |  |
|  | **Additional editor comments** |  |
| 2. | Please, carefully address the earlier queries of Reviewer 4, which were not addressed in the revised version of the manuscript (although this was stated in the authors reviewers reply). | Thank you. This has been addressed as shown below. |
|  | **Reviewer 4 comments** |  |
|  | Experience and Knowledge level of the RTTs involved (Aged 27-49 years) is missing | Thank you for this comment. This has been revised in the result section of the revised manuscript line 133-135 |
|  | Internal consistency reliability test and coherence of the Thematic analysis is missing | Thank you for this comment.  I understand that reliability test and coherence is very important. However, in qualitative studies, this is described as how the trustworthiness of the study will be realized. There are four criteria to consider; credibility, transferability, confirmability and dependability. This was not included in the earlier version of the manuscript, but due to its importance, it has been included in the revised manuscript. Line 245-255 |
|  | **Reviewer 1** |  |
|  | Dear authors,  I do not have any further comments to the article.  Kind regards | Thank you so much |
|  | **Reviewer 2** |  |
|  | The discussion needs to be revised according to Reviewer 2 comments.  Standardize the type of parentheses in the Bibliographic References, straight or curved (according to the rules of the publication). The Discussion could be more accomplished, expression as "This study revealed significant differences in participants’ understanding of the thermoplastic mask preparation process" (l248), are "too strong". | Thank you so much for this observation. This has been revised in a revised manuscript line 225 |
|  | There is a need for these studies in their conclusions to contain implications and to contribute to the improvement of practices. | Thank you for this observation. We have revised the conclusion section to include the implications of the study and how the findings will improve the practice.  Line 260-271 |
|  | **In addition to the manuscript** |  |
|  | Study limitation section | We have added this section in our revised manuscript to clear any doubts from our readers as also asked by reviewer 2. Line 256-259 |

To our knowledge, we have addressed all the comments and questions raised by the reviewers. We, therefore, kindly ask you to reconsider our manuscript for publication in this very good journal.

Yours Sincerely

Furahini Yoram,

Corresponding author.

Email: [furahiniyoram@gmail.com](mailto:furahiniyoram@gmail.com)

Contact: +255756470122
